# Rift Valley fever, Mauritania, 2020: Lessons from a one health approach

**DOI:** 10.1016/j.onehlt.2022.100413

**Published:** 2022-06-27

**Authors:** Yahya Barry, Ahmed Elbara, Mohamed Abdallahi Bollahi, Ahmed B. Ould El Mamy, Mokhtar Fall, Abdellahi Diambar Beyit, Mariem Seyidna Khayar, Ba Aliou Demba, Mohamed Limine Haki, Ousmane Faye, Ludovic Plee, Etienne Bonbon, Baba Doumbia, Elena Arsevska, Catherine Cêtre-Sossah

**Affiliations:** a Office National de Recherches et de Développement de l'Élevage et du Pastoralisme (ONARDEP), Nouakchott, Mauritania; b Institut National de Recherches en Santé Publique (INRSP), Nouakchott, Mauritania; c World Health Organization, Nouakchott, Mauritania; d UCAD, Université Cheikh Anta Diop, Dakar, Senegal; e Food and Agriculture Organization of the United Nations (FAO), Rome, Italy; f Ministère du Développement Rural, Nouakchott, Mauritania; g CIRAD, UMR ASTRE, F 34398 Montpellier, Cedex, France; h ASTRE, Montpellier University, CIRAD, INRAE, Montpellier, France

## Abstract

A new outbreak of Rift Valley fever (RVF) occurred in Mauritania from September to November 2020, involving 78 reported human cases and 186 reported animal cases. Eleven out of the 13 regions of the country were affected by the epidemic, with the highest number of both human and animal cases in Tagant, Assaba and Brakna regions. The most affected animal species in this outbreak was camels, followed by small ruminants. Among the 10 mosquito species caught, 7 species, *Culex poicilipes, Cx. quinquefasciatus, Cx. antennatus, Cx. univitattus, Aedes vexans, Mansonia africana* and *Ma. uniformis*, are known to be involved in the transmission of RVF virus. Phylogenetic analyses based on the partial NSs gene revealed close proximity between the human/animal Mauritania 2020 viral strains and the Mauritania 2015/Niger 2016 strains, suggesting re-emergence of the RVF virus in the country since the last reported outbreak in 2015.

## Highlights

• New outbreak of Rift Valley fever (RVF) in Mauritania in 2020, 78 human and 186 animal reported cases.
• Eleven regions of the country were affected by the epidemic, with the highest number of human and animal cases in Tagant, Assaba and Brakna regions.
• The most affected animal species were dromedaries followed by small ruminants.
• Close proximity of the human/animal Mauritania 2020 viral strains with the Mauritania 2015/Niger 2016 strains based on NSs phylogenetic analysis

## 1 Introduction

Humans are infected by Rift Valley fever virus (RVFV; family *Phenuiviridae*, genus *Phlebovirus*) [1] through contact with blood or organs during handling or slaughter of infected animals, consuming contaminated meat or contaminated raw milk. Animal infections are caused by the bite of infected mosquitoes, mainly *Aedes* spp. and *Culex* spp. [2,3] leading to a high mortality rate among young ruminants and to numerous abortions of pregnant females. Symptoms observed in human infections range from influenza-like illness to severe symptoms including haemorrhages, encephalitis, hepatitis, ocular complications and fatal outcomes (https://www.cdc.gov/westnile/dengue/riftvalleyfever/chikungunya).

West Africa and particularly Senegal and Mauritania are severely affected by RVF. Several outbreaks have occurred since RVF was first described along the Senegal River in Mauritania at the end of the 1987 rainy season with an estimated In 12 regions defined as at risk of RVF occurrence in the country (disease already reported in the region, proximity and nature of human deaths [[4], [5], [6]]. This first outbreak led to the creation of an active surveillance system that allowed the detection of several animal cases in Mauritania, Senegal, and other West African countries [[7], [8], [9]].

Further, RVFV has been repeatedly isolated in different mosquito species during inter epidemic periods in Senegal, Burkina Faso, Nigeria [10]. In 1998, an outbreak of RVF occurred in Aïoune El Atrouss in the region of Hodh El Gharbi in the south-eastern part of Mauritania resulting in 300 to 400 human cases including 6 deaths [11]. Five years later, in 2003, extended regions of the south, southeast, and central regions of Mauritania experienced RVF outbreaks with 25 confirmed human cases, including 16 cases of haemorrhagic forms and 4 deaths, in the south (Trarza, Brakna, Gorgol), southeast (Assaba), and central (Tagant) regions of the country [12]. The northern desert region of Mauritania remained free of RVF until October 2010, when an RVF outbreak was reported in the 2 regions of Adrar and Inchiri, with a total of 70 human cases, including 13 deaths [13]. This outbreak was followed by two more epidemics, the first in 2012 [14,15] with 36 confirmed human cases including 19 deaths, and the second in 2015 with 57 confirmed human cases including 12 deaths distributed in several regions (Hodh El Gharbi, Assaba, Brakna, Trarza, and Gorgol, Tagant) [16,17]. During epidemics that have occurred since 1998 in Senegal and Mauritania, at least 5 species have been shown to be vectors of RVF of the Aedes, Culex and Mansonia genera, *Culex poicilipes* and *Cx. quiquefasciatus, Aedes vexans, Mansonia africana* and *Ma. uniformis* [[18], [19], [20], [21]].

Following the appearance of symptoms of haemorrhagic fever on September 4, 2020, the first human case of RVF was diagnosed on September 14, 2020. Subsequently, the blood of 235 suspected human cases of RVF was sampled between September 13 and November 18, 2020. Seventy-eight human cases were tested positive for RVFV using RTqPCR and were officially reported to the World Health Organization (WHO) [22]. Among the 78 human cases, there were 25 deaths, but the true number was probably much higher. This means the 2020 Mauritania RVF outbreak ranks as one of the most severe in terms of the mortality rate.

In parallel to human cases, severe cases in camels and waves of abortions in small ruminants and cattle were widely distributed i.e. in 11 regions of Mauritania: Assaba, Brakna, Guidimaka, Hodh Ech Chargui, Hodh El Gharbi, Inchiri, Nouakchott, Tagant and Trarza compared to the previous outbreaks in 1987, when only the southern part of the country (Trarza, Brakna) was affected and to the 2010 outbreak, when only the northern part of the country was affected (Adrar, Inhiri). The first animal case was sampled on September 16, 2020 and was confirmed as RVF positive on September 21, 2020 by RVFV specific IgM ELISA. This animal was a bovine owned by the human index case, who lived in the same village in the region of Assaba. In total, 186 animals, among which 94 camels, 89 small ruminants and 3 bovines, were diagnosed positive either by RVFV specific IgM ELISA or by RVFV specific RT-PCR genome detection [23].

Here we describe the 2020 Mauritania RVF outbreak including the results of serological and molecular investigations of human and animal samples as well as some entomological findings.

## 2 Materials and methods

### 2.1 Case definition

A suspected human case of RVF is defined as a patient suffering from axillary temperature > 37.5 °C for 48 h with or without haemorrhagic signs (cutaneous bleeding, bleeding from mosquito bite sites, epistaxis, gingival, or other bleeding) or neurological signs (exhaustion, myalgia, headache, nausea/vomiting, diarrhoea), jaundice, or retinitis during the rainy season (August to December), the period known to be at risk of RVF occurrence due to favourable climatic factors (rainfall, abundant mosquitoes).

A suspected animal case of RVF is defined as an animal that aborted during a wave of abortions by pregnant animals in one herd or the death of a young animal (<2 months of age) with or without fever observed during the rainy season (August to December), a period known to be at risk of RVF occurrence due to favourable climatic factors (rainfall, abundant mosquitoes).

A confirmed RVF human or animal case is defined as a laboratory-diagnosed case of acute or recent RVFV infection tested positive by serology using specific RVFV IgM ELISA and/or by genome detection using RVFV specific RT-PCR.

### 2.2 Collection of human and animal samples

The participants were selected based on a medical consultation they had at the closest health medical centres and were considered as suspected RVF patients based on their clinical signs. Each participant was interviewed using a standard questionnaire that included information on age, gender, region of residence, as well as the sample collection date. Human blood samples were collected from a total of 235 patients suspected of having RVF. A total of 640 blood samples were collected from 360 small ruminants and bovines, and 280 camels suspected of having RVF (Table 1).

**Table 1:** Results of the serological and molecular biological investigation of suspected animal cases of RVF. Blood samples were taken between September 16 and October 24, 2020, in Mauritania. IgM and RT-PCR tests were used to detect the 2020 RVF outbreak in small ruminants/bovine and camels respectively. Number, n°.

Region	Small ruminant and bovine samples, n°. (%)		Camel samples, n°. (%)		Total n° of positive animals/total n° of samples, n° (%)
	Number sampled	IgM positive	Number sampled	RT-PCR positive	Total n° of samples
Adrar	14	1 (7%)	0	0	1/14 (7%)
Assaba	70	7 (10%)	10	4 (40%)	11/80 (14%)
Brakna	86	32 (37%)	25	4 (16%)	36/111 (32%)
Dakhlet Nouadhibou	12	1 (8%)	0	0	1/12 (8%)
Guidimakha	30	1 (3%)	0	0	1/30 (3%)
Hodh Ech Chargui	37	9 (24%)	77	26 (34%)	34/114 (30%)
Hodh El Gharbi	35	5 (14%)	34	12 (35%)	17/69 (25%)
Inchiri	0	0	23	4 (17%)	4/23 (17%)
Nouakchott	0	0	31	8 (26%)	8/31 (26%)
Tagant	33	20 (61%)	32	18 (56%)	38/65 (58%)
Trarza	43	16 (37%)	48	18 (37%)	34/91 (37%)
	360	92	280	94	186/640 (29%)

A national surveillance plan for early detection of RVF had already been set up in 12 regions in Mauritania defined as at risk of RVF occurrence, i.e. the disease had already been reported in the region, the proximity and nature of the water in the Senegal River, lakes, large wadis or Tamourts, more or less persistent ponds, irrigated perimeters, wetlands without open water),. This led to an additional collection of blood samples during the 3 months of the rainy season (September–December) in 2020, which corresponded to the period of the 2020 RVF outbreak reported in this paper. This serology monitoring campaign resulted in 28 sentinel herds containing an average of 15 animals/herd being sampled between September 25 and September 30, 2020 (Table 2).

**Table 2:** Results of the serological monitoring of sentinel herds for RVF in small ruminants between 25 and 30 September 2020, Mauritania.

Region	Site	Number of sampled animals	Number of RVF IgM positive animals	Percentage of RVF IgM positive animals per site	Mean percentage of RVF IgM positive animals per region
Adrar	1	14	1	7.14	3.7
	2	13	0	0.00	
Assaba	3	15	1	6.67	6.9
	4	14	1	7.14	
Brakna	5	15	0	0.00	7.02
	6	15	1	6.67	
	7	15	0	0.00	
	8	12	3	25.00	
Dakhlet Nouadhibou	9	12	1	8.33	8.33
Gorgol	10	15	0	0.00	0
	11	15	0	0.00	
Guidimakha	12	15	0	0.00	0
	13	15	0	0.00	
	14	15	0	0.00	
Hodh ech Chargui	15	13	1	7.69	5.0
	16	13	0	0.00	
	17	14	1	7.14	
Hodh el Gharbi	18	12	2	16.67	11.43
	19	15	2	13.33	
	20	8	0	0.00	
Inchiri	21	13	0	0.00	0
	22	15	0	0.00	
Tagant	23	15	4	26.67	60
	24	15	14	93.33	
Tiris Zemmour	25	6	0	0.00	0
Trarza	26	15	0	0.00	2.27
	27	14	0	0.00	
	28	15	1	6.67	

### 2.3 RVF laboratory diagnosis

RVF diagnosis was performed as follows: RVFV specific RTqPCR was used for human blood samples; RVFV specific RTqPCR for camels and IgM ELISA for small ruminants/bovines. Human RVFV was diagnosed at INRSP with, as a first step, RNA extraction using the QIAamp viral RNA kit (Qiagen, USA) followed by a RT-qPCR using the RealStar® Rift Valley Fever Virus RT-PCR Kit 1.0 (Altona Diagnostics GmbH, Germany) in a Rotor-Gene Q system (Qiagen USA) following manufacturer's instructions.

Animal RVF was diagnosed in samples of camel blood through the detection of RVFV specific genome with, as a first step, RNA extraction using the Nucleospin RNA virus kit (Macherey-Nagel, Germany) followed by RT-qPCR [24] in a Mic RealTime PCR cycler (Bio Molecular Systems, Australia), whereas the detection of RVFV specific IgM ELISA was done with the ID Screen® Rift Valley Fever IgM Capture kit (ID.vet, Grabels, France) in samples of small ruminants/bovines blood.

### 2.4 Sequencing and phylogenetic analysis

Eight out of the 78 human samples and 6 out of the 94 animal samples that were RTqPCR–positive for RVFV were used to sequence the NSs gene of the S segment by full length S-Segment PCR amplification [25]. To compare the genetic relatedness of the sequenced viruses, phylogenetic analyses were performed against a panel of 157 published NSs RVFV nucleotide sequences. Before phylogenetic inference, datasets and multiple sequence alignments were thoroughly checked to eliminate misalignments and ensure correct framing of the coding sequences. Sequences were aligned by ClustalW, edited using MEGA X software [26]. The evolutionary history was inferred using the maximum likelihood (ML) method and general time reversible model (GTR) [27] implemented in the above mentioned MEGA X. The 100% nucleotide identity observed for the sequenced samples led us to choose one strain per host species, namely camel case 13 and human case 7 for further phylogenetic analysis.

### 2.5 Sampling, identification and storage of adult mosquitoes

During the epidemics, state spraying with insecticides was carried on two occasions, which may have affected the number of mosquitoes trapped. Knowing that spraying insecticide reduces the mosquito populations, we targeted villages where the inhabitants were still reporting the presence of large numbers of mosquitoes. Two mosquito traps were used: CDC light traps (LTs) (CDC miniature light trap, BioQuip Products, Inc., Rancho Dominguez, CA) with CO2 and intra-household aspiration at the rate of one night of capture from 7 pm to 7 am near the herds or near a marsh. Adult mosquitoes were morphologically identified using the P.F. Mattingly (1971) and A.G. Fall (2013) [28,29] keys and pooled according to species, sex, blood meal status (engorged or unfed female), date of capture and the type of trap used.

### 2.6 Statistical analysis

Descriptive statistical analysis was used to characterize the key demographic variables of human RVF cases. We fitted a multivariate binomial logistic model to predict the outcome (confirmed or unconfirmed case of RVF) using the predictor variables, selected using a backward selection based on the lowest Akaike information criterion (AIC) from a full model including all possible predictor variables (region, age, gender, occupation, hospitalization, outcome, general lethargy, general fever clinical signs, musculoskeletal, digestive, haemorrhagic, neurological and ocular clinical signs). Regression results were considered significant for *p*-values ≤0.05. All analyses were performed using R software (version 4.1.2) [30].

### 2.7 Ethics

No endangered or protected species were involved in the surveys A signed consent form was obtained from each patient for blood donation. Animal samples were taken by ONARDEL as part of its government mandate to conduct livestock animal monitoring and surveillance programmes for veterinary and zoonotic pathogens while respecting all relevant national as well as international regulations and fundamental ethical principles. Farmers in each zone gave their verbal consent to be included in the study. Verbal consent to sample blood in a herd was obtained from the owner of the herd after information was provided in the local language.

## 3 Results

Between September 4 and November 18, 2020, Mauritanian authorities reported a total of 78 confirmed cases of human RVF out of 235 suspected cases of human RVF and 156 confirmed cases of animal RVF out of 640 suspected cases of animal RVF.

### 3.1 Human cases

**Fig. 1:**
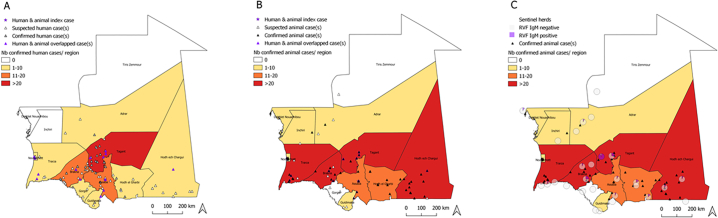
Distribution of human and animal RVF cases in Mauritania, 2020. A. Humans, B. in Animals. C) results from serological monitoring of sentinel herds. The choropleth map shows the number of confirmed A) human and B) animal cases per region, respectively.

The 235 suspected RVF cases originated from 53 villages geographically distributed in 10 regions, Adrar, Assaba, Brakna, Gorgol, Guidimaka (at the Senegal border), Hodh Ech Chargui, Hodh El Gharbi (at the border with Mali), Nouakchott, Tagant and Trarza, with different occurrence rates (Fig. 1A). The region of Tagant was the most severely affected (38/78, 49%) with two districts in the region, Tidjikja and Moudjeria, more affected than others [22]. The Index case was a 70-year-old male trader from the Kiffa Moughataa (Assaba region), who showed general, neurological, haemorrhagic and digestive clinical signs with no specific RVF clinical signs that started on September 4, 2020. He was diagnosed as RVF positive and the diagnosis was confirmed by a laboratory on September 14, 2020.

Males (*n* = 64; 82%) were the most affected, the majority being farmers (*n* = 47; 60%).

All age groups were affected, the majority being between 25 and 44 years of age (*n* = 32; 41%), followed by those 15 to 24 years old (*n* = 25; 32%). The majority of patients had lethargy (*n* = 37; 47%), fever and gastrointestinal clinical signs (*n* = 52; 67% respectively) (Table 3), and the majority of patients recovered after infection (*n* = 53; 68%).

**Table 3:** Description of the confirmed cases of human RVF during the RVF outbreak 2020, Mauritania.

Characteristics	Confirmed cases of RVF (%)
Region	
Adrar	1 (1)
Assaba	18 (23)
Brakna	10 (13)
Gorgol	1 (1)
Guidimaka	2 (3)
Hodh ech Chargui	1 (1)
Hodh el Gharbi	3 (4)
Nouakchott	2 (3)
Tagant	38 (49)
Trarza	2 (3)
Sex	
Female	14 (18)
Male	64 (62)
Age group (years)	
<5	2 (3)
5–14	8 10)
15–24	25 (32)
25–44	32 (41)
>44	11 (14)
Occupation	
Farmer	47 (60)
Housekeeper/housewife	7 (9)
Pupil	13 (17)
Trader	6 (8)
Unemployed	5 (6)
Hospitalization	
Yes	70 (90)
No	8 (10)
Outcome	
Dead	25 (32)
Alive	53 (68)
Clinical signs	
General	
Headache/Fever	52 (67)
Asthenia/Lethargy/Weakness	37 (47)
Musculoskeletal	
Myalgia/Arthralgia	23 (29)
Digestive	
Nausea/Vomiting/Diarrhoea/Abdominal pain/Dysphagia	52 (67)
Haemorrhagic	
Petechia/Purpura/Melena/Epistaxis/Gingivorrhagia/Icterus	23 (29)
Neurologic	
Confusion/Hiccups/Vertigo	30 (38)
Ocular	
Retroorbital pain/Ocular disorder	2 (3)

We fitted a binomial logistic model (estimated using ML) to predict the result (confirmed or unconfirmed case of RVF). The model's explanatory power is substantial (Tjur's R2 = 0.41). Within this model, the effect of gender [Male] was found to be statistically significant and positive (beta = 1.47, 95% CI [0.54, 2.51], *p* = 0.003; Std. beta = 1.47, 95% CI [0.54, 2.51]) just like the effect of outcome [Dead] (beta = 3.12, 95% CI [1.87, 4.73], *p* < 0.001; Std. beta = 3.12, 95% CI [1.87, 4.73]), and the effect of digestive clinical signs [Yes] (beta = 2.08, 95% CI [0.43, 4.29], *p* = 0.029; Std. beta = 2.08, 95% CI [0.43, 4.29]) (Table 4).

**Table 4:** A multivariate logistic regression model with the significant (p-value ≤0.05) variables predictors of Rift Valley fever cases in humans in the 2020 epidemics in Mauritania.

Variable	Level	Beta	95% Confidence Interval (CI)	p-value
Intercept	–	−6.66	−11.54, −2.14	*p* = 0.005
Region	Assaba	1.82	−2.04, 5.82	0.353
	Brakna	0.52	−3.35, 4.50	0.791
	Dakhlet Nouadhibou	−14.70	−617.70, 48.10	0.993
	Gorgol	2.37	−2.47, 7.24	0.330
	Guidimaka	2.74	−1.60, 7.25	0.219
	Hodh ech Chargui	1.35	−5.96, 3.01	0.545
	Hodh el Gharbi	1.31	−2.71, 5.47	0.522
	Inchiri	−13.63	−585.32, 45.46	0.993
	Nouakchott	−0.77	−4.82, 3.36	0.706
	Tagant	2.40	−1.42, 6.35	0.214
	Trarza	1.39	−2.77, 5.66	0.510
Gender	Male	1.47	0.54, 2.51	0.003
Hospitalization	Yes	0.88	−0.19, 2.01	0.115
Outcome	Dead	3.12	1.87, 4.73	< 0.001
General lethargy clinical signs	Yes	0.67	−0.18, 1.56	0.130
Digestive clinical signs	Yes	2.08	0.43, 4.29	0.029
AIC	220.9			

### 3.2 Animal cases

The 640 animals that were suspected of having RVF (360 small ruminants and bovines, and 280 camels) were distributed in 11 regions: Adrar, Assaba, Brakna Dakhlet Nouadhibou, Guidimaka, Hodh Ech Chargui, Hodh El Gharbi, Inchiri, Nouakchott, Tagant and Trarza with different occurrence rates (Fig. 1B).

Of the 360 RVF suspected cases in small ruminants (330 animals) and bovines (30 animals), 92 cases were confirmed by the laboratory. These cases were distributed across 9 regions (Adrar, Assaba, Brakna, Dakhlet Nouadhibou, Guidimaka, Hodh ech Chargui, Hodh el Gharbi, Tagant and Trarza) out of the 13 regions of Mauritania. The highest number of RVF cases in small ruminants was reported in the region of Brakna (*n* = 32; 35%), followed by Trarza (*n* = 16; 17%) and Tagant (*n* = 20; 22%) regions (Table 1, Fig. 1B). Out of the 30 RVF suspected bovine cases, 3 were confirmed as positive for RVF by the laboratory, the 3 cases were located in two villages in the region of Assaba, (Table 1, Fig. 1B).

Out of the 280 camels suspected of having RVF, 94 cases were confirmed by the laboratory using the RVF specific RT-PCR method. The 94 cases were located in eight regions Assaba, Brakna, Hodh ech Chargui, Hodh el Gharbi, Inchiri, Nouakchott, Tagant and Trarza out of the 13 regions of Mauritania. The most cases were reported in the regions of Hodh ech Chargui (*n* = 26; 28%), Tagant (*n* = 18; 19%) and Trarza (n = 18; 19) (Table 1, Fig. 1B).

**Fig. 2:**
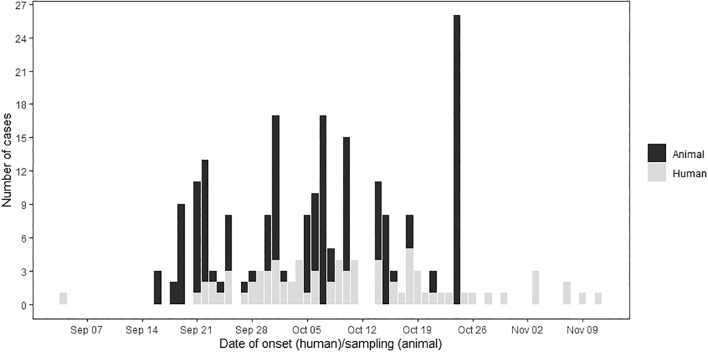
Distribution of the cumulated confirmed human and animal RVF cases based on the date of onset in humans and date of sampling in animals, between 4th September and 15th November 2020 in Mauritania.

During the 2020 RVF outbreak, 30 (30/78; 38%) of the human cases of RVF human were reported in the same villages as the cases of animal RVF and 37 cases (37/78; 47%) within a 10-km radius of the animal cases, specifically in the regions of Assaba, Brakna, Hodh ech Chargui, Nouakchott, Tagant and Trarza (Fig. 2B).

### 3.3 Temporal distribution of RVF human and animal cases

From September 4, 2020 to November 18, 2020, a total of 78 human cases were confirmed by RVF specific RTqPCR and RVF specific IgM ELISA; 186 animal cases were confirmed as RVF positive (94 camels by RVF specific RTqPCR and 92 small ruminants and bovines by RVF specific IgM ELISA. The epidemic curve concerning animals peaked on October 24, 2020, when 24 cases were confirmed. There was no real peak in human cases of RVF as they occurred throughout the period of the outbreak. The window between September 19, 2020 and October 24, 2020 included most human and animal cases. The last confirmed case of RVF occurred in week 28 (Fig. 2). The first and the last confirmed cases of the RVF outbreak were human.

### 3.4 RVF serological investigations in sentinel herds

The results of serological monitoring between September 25 and September 30, 2020 of 28 sentinel herds in 12 regions at risk of RVF occurrence showed a low level of anti-RVF specific IgM prevalence. The highest prevalence was observed in two regions, (i) the region of Tagant with one sentinel herd harbouring 14 IgM positive animals out of the 15 animals tested and a second sentinel herd with 4 IgM positive animals out of the 5 tested animals) and (ii) the region of Brakna with one sentinel herd with 3 IgM positive animals out of the 12 animals tested (Fig. 1C, Table 2). No RVF virus was isolated.

### 3.5 Mosquito diversity

A total of 828 mosquitoes belonging to 4 genera and 10 species were collected between October 16, 2020 and October 30, 2020, in the 18 target sites using CDC-type light traps. No mosquitoes were collected in 6 of the 18 traps. Mosquitoes belonging to the genus *Culex* were the most abundant with 59,7% caught in 11 catches followed by mosquitoes from the genus *Anopheles* spp.*, Mansonia and Ae. vexans* (Table 5). Seven species namely *Culex antennatus, Cx. poicilipes, Cx. quinquefasciatus, Cx univitattus*, *Aedes vexans, Mansonia africana and Ma. uniformis* known to be involved in the transmission of RVF virus *18,31*] were collected and morphologically identified and represented 58,45% of the total number of mosquitoes. The regions where the most abundant population of mosquitoes was caught were, in decreasing order Brakna, Hodh El Gharbi, Hodl Ech Chargui, Trarza, which coincides with the regions with the highest number of animal and human cases except for the region of Tagant where low numbers of mosquitoes were caught despite the high number of animal and human cases observed there.

**Table 5:** Diversity of trapped mosquitoes according to the location and species. Cx: Culex, Ma: Mansonia.

			Species												
			Culex poicilipes^a^	Cx. quinquefasciatus^a^	Cx. bitaeniorhynchus	Cx. univitattus^a^	Cx. antennatus^a^	Cx. aurantapex	Cx. sitiens	Cx. male spp.	Mansonia uniformis^a^	Ma. africana^a^	*Aedes vexans* arabiensis^a^	Anopheles spp.	Total
Wilaya	Moughataa	Locality													
Trarza	Rosso	Rosso	2	16	3	0	0	0	0	34	26		5	3	89
Trarza	Keurmacène	Keurmacène	0	2	0	0	0	5	2	1	4	5		5	24
Trarza	Rkiz	Mare Rkiz	0	0	0	0	0	0	0	0	0	0	0	0	0
Tagant	Moudjeria	Nbeika	0	9	0	0	0	0	0	16	0	0	0	0	25
Guidimaka	Ghabou	Koumba ndao	0	12	0	0	0	0	0	25	0	0	0	0	37
Guidimaka	Selibabi	Moudji	0	0	0	0	0	0	0	0	0	0	0	0	0
Gorgol	Mbout	Foum Gleita	0	0	0	0	0	0	0	0	0	0	0	0	0
Gorgol	Kaédi	Lexeiba		15	0	0	0	0	0	0	0	0	0	0	15
Gorgol	Kaédi	Ganki	0	10	0	0	0	0	0	0	11	0	1	1	23
Brakna	Boghe	Boghe	0	0	0	0	0	0	0	0	0	0	0	0	0
Brakna	Aleg	Aleg	58	58	7	3	4	0	0	43	12		12	52	249
Brakna	Maal	Maal	0	0	0	0	0	0	0	0	0	0	0	0	0
Hodh El Gharbi	Aioune	Gounguel	8	12	0	0	0	0	0	17	12	0	15	6	70
Hodh El Gharbi	Aioune	Aioune	0	19	0	0	0	0	0	13	10	0	5	12	59
Hodh El Gharbi	Kobeni	Kobeni	9	21	5		0	0	0	15	4	0	2	10	66
Hodh Ech Chargui	Néma	Mahmouda	9	14	7	4	5	0	0	11	31	0	20	40	141
Hodh Ech Chargui	Amourj	Amourj	0	0	0	0	0	0	0	0	0	0	0	0	0
Assaba	Kiffa	Kiffa	0	0	0	0	0	0	0	0	0	0	19	11	30
		Total	86	188	22	7	9	5	2	175	110	5	79	140	828

a Mosquito species which have demonstrated vector competence in nature or in the laboratory.

### 3.6 Genetic diversity

**Fig. 3:**
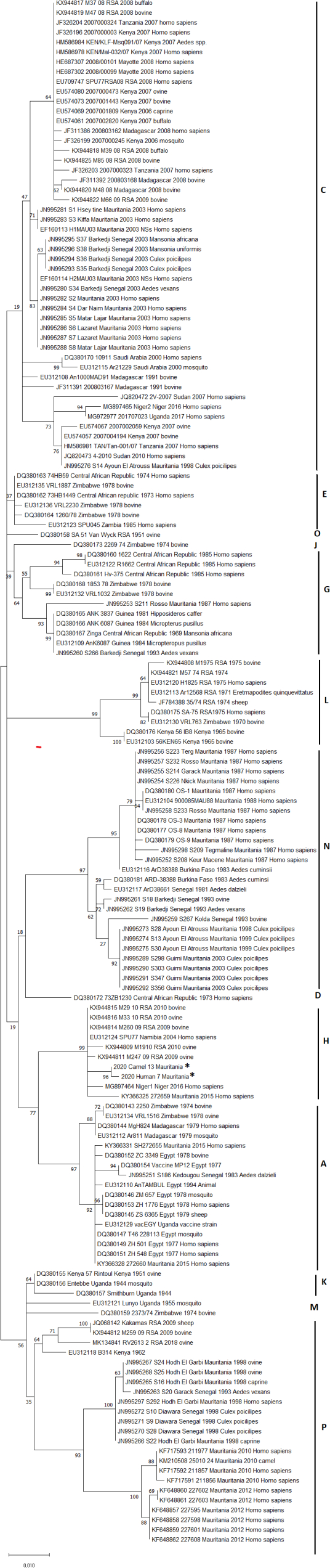
Phylogenetic tree derived from nucleotide sequence data of the S segment, partial NSs gene. The tree with the highest log likelihood (-2460.73) is shown. The percentage of trees in which the associated taxa clustered together is shown next to the branches. Initial tree(s) for the heu-ristic search were obtained automatically by applying Neighbor-Join and BioNJ algorithms to a matrix of pairwise distances estimated using the Maximum Composite Likelihood (MCL) approach, and then selecting the topology with superior log likelihood value. A discrete Gamma distribution was used to model evolutionary rate differences among sites. (5 categories (+G, parameter = 0.3199)). The rate variation model allowed for some sites to be evolutionari-ly invariable ([+I], 35.26% sites). The tree is drawn to scale, with branch lengths measured in the number of substitutions per site. Classification of the isolates followed the lineage terminology of Grobbelaar et al., 2011 [46]. The GenBank accession numbers for the NSs gene of the virus S segment are ON052829(2020 Camel 13 Mauritania) and ON052830 (2020 Human 7 Mauritania).

RNA was extracted from 14 RVF RT-PCR positive samples originating either from humans (*n* = 6) or from animals (*n* = 8) followed by the amplification and sequencing of the S segment. No differences in amino acids (aa) were found between the S segments analysed. Since only partial S segments, specifically from the NSs gene, were available for West Africa (Senegal and Mauritania) in GenBank, the phylogenetic analysis was based on the NSs gene of the RVFV S segment. One hundred percent identity was found between the human and the camel sequences that were amplified during the outbreak of RVF in Mauritania in 2020. The sequences consistently clustered with the West African strains, specifically found in Senegal and Mauritania in 2013 and 2015, as well as Niger in 2016, in the genetic lineage H as the North African Egyptian strains (Fig. 3).

## 4 Discussion

The 2020 epidemic of RVF that occurred in Mauritania resulted in 25 human deaths and at least 219 cases of domestic animals, especially of camels. As described in previous epidemics, men were more affected than women, animal herders being the most affected. The most affected age-groups were young adults (15 to 24 years of age) followed by adults aged between 25 and 44 years of age. In the affected villages, high abundance of mosquitoes was observed linked with a lot of temporary marshes. The two main ways of transmission of RVF between humans, direct contact when slaughtering animals versus mosquito bites are difficult to distinguish but both routes are likely.

In the outbreak in 2010, unexpectedly only the 3 desert regions of Adrar, Inchiri and Dakhlet Nouadibou located the northern part of Mauritania were affected for the first time with a high mortality rate of camels combined with the small ruminant and human cases due to abnormal rainfall events [13,31] in contrast to the outbreak in 2012 when only 7 regions known to be at risk of RVF were affected (Tagant, Hodh Ech Chargui, Hodh El Gharbi, Assaba, Brakna, Trarza and Nouakchott) with 41 human cases and 13 deaths due to abortions by small ruminants [14]*.* The outbreak of RVF in Mauritania in 2015, with 184 suspected cases, and 57 confirmed cases including 12 deaths occurred in regions similar to those affected in the outbreak in 2020 [17].

The severity of the clinical cases in the RVF outbreak of 2020 and their large spatial distribution was remarkable with a large number of human and animal deaths and specific clinical signs that were observed for the first time vaccinsuch as blindness and haemorrhagic syndrome in 83 out of 357 of the camels. All animal species were affected, in particular in the Assaba region, where the outbreak started with a cow that aborted early on September 4, 2020 which coincided with the clinical signs observed in the index human case. Animal mobility to get pasturages, livestock markets and the the period in the year when religious ceremonies are celebrated on the top of abnormal climatic events may play a key role in the occurrence and the severity of RVF outbreaks [32,34].

Based on maximum likelihood trees, our data showed that Mauritanian 2020 strains clustered with Mauritanian 2015 and Niger 2016 strains [33] as well as with South African 2009–2010 strains, closely related to Egyptian strains, suggesting close proximity between the 2015 and 2020 RVF epidemics that occurred in Mauritania. The origins of the 2020 outbreak are likely due to animal mobility between neighbouring countries as commercial animal exchanges exist between countries, although the last reported outbreak of RVF in Senegal was in 2013 [9] and unfortunately no sequences of the 2013 outbreak were available in the database to allow the comparison and to suggest either the introduction of the isolates from Senegal, or from Egypt to Mauritania, which are both neighbouring countries where animal mobility is likely [34].

Among the 10 species identified, seven species, *Culex antennatus*, *Cx. poicilipes, Cx. quinquefasciatus*, *Cx univitattus*, *Aedes vexans, Mansonia africana and Ma. uniformis*, are known to be involved in the transmission of the RVF virus [31] with a low yield of *Aedes vexans* as primary vectors (79/828) in the present study. The biting preferences of mosquitoes may provide insight as to the predominant method of transmission between humans and animals. The trophic preference of mosquitoes depends mainly on the type of environment and the season where they were collected (urban versus rural) and the availability of feeding hosts. In Kenya, among the different species collected during a 2007 post epidemic study, *Cx. quinquefasciatus* was found to be the only species gorged on human blood [35]. Few years later, Stoek et al. [36] confirmed the human trophic preference of *Cx. quinquefasciatus* and *Cx poicilipes*. On the other hand, in 1993, in Egypt, *Cx. antennatus* preferred to feed on cattle [37]. *Aedes vexans* is known for its opportunistic feeding behavior, although it prefers to feed on mammals, especially horses (primary hosts) and ruminants (secondary hosts) [38]. *Ma. uniformis* fed mainly on sheep (38%), frogs (13%), duikers (8%), cattle (4%), goats (4%) [39].Serological monitoring of sentinel herds started September 25, 2020, and revealed high circulation of RVF in the Tagant region where 60% of the animals tested RVF IgM positive. The timely creation of sentinel herds is a key factor in the efficiency of an early warning system aimed at preventing an epidemic in humans. The establishment of sentinel herds in 2020 was delayed linked to administrative formalities and partnership financial issues. The serological monitoring of sentinel herds in the rainy season is crucial as it enables detection of early circulation of RVFV. Early detection of RVF specific IgM antibodies in sentinel herds allows communication between human and animal health communities concerning the measures to be taken before RVF reaches the human compartment, i.e. through protective measures and informing the human populations.

In the framework of animal health surveillance management, it is recommended to use national or regional prediction models to enable early warning [[40], [41], [42]]. It is crucial to install the animal sentinel herds at the start of the rainy season in order to strengthen syndromic surveillance of arboviruses among breeders, herders, and farmers and to raise awareness among public health professionals (medical doctors, pharmacists, nurses in local health centers) concerning the definition of cases of arboviral and haemorrhagic fevers. The fact animal cases of RVFV are widespread in the southern part of Mauritania, point to a potentially high risk of amplification of the virus linked to the migration of herds of domestic animals which share the same pasture and and human populations who share the same transhumance practices [4].

Efforts need to be united and strengthened to better control the emergence of RVF outbreaks in Mauritania by (i) implementing common RVF dedicated communication actions between the public health and the veterinarian sectors a few weeks before the beginning of the rainy season, (ii) forecasting outbreaks by establishing sentinel herds at the right time to predict the arrival of potential RVF outbreaks as early as possible by using risk-mapping modeling and (iii) by vaccinating animals in target areas to prevent human infection and to limit widespread viral amplification [43]*.* In endemic area like Mauritania, it is important to use a safe vaccine that requires only one vaccination [44,45] to break the Rift Valley fever cycle and to prevent the possible spread of RVFV from one continent to another through animal mobility.

## Author contributions

Conceptualization, Data curation, Formal analysis, Methodology: ADB, AE, ALB, MAB, MSK, YB

Investigation: MF, LP, EB, CCS, BD, AOM

Data curation, Supervision, Validation, Writing-original draft, Wrting-reviwe and editing: EA, CCS, YB

## Declaration of Competing Interest

The authors have no conflict of interest to declare.
